# Treating Smoking Dependence in Depressed Alcoholics

**Published:** 2006

**Authors:** Nassima Ait-Daoud, Wendy J. Lynch, J. Kim Penberthy, Alison B. Breland, Gabrielle R. Marzani-Nissen, Bankole A. Johnson

**Affiliations:** Nassima Ait-Daoud, M.D., is an assistant professor; Wendy J. Lynch, Ph.D., is an assistant professor; J. Kim Penberthy, Ph.D., is an assistant professor; Alison B. Breland, Ph.D., was an assistant professor; Gabrielle R. Marzani-Nissen, M.D., is an assistant professor; and Bankole A. Johnson, D.Sc., M.D., Ph.D., is an alumni professor and chairman, all in the Department of Psychiatry and Neurobehavioral Sciences, University of Virginia, Charlottesville, Virginia. Alison B. Breland, Ph.D., currently is an adjunct instructor in the Department of Psychology, Virginia Commonwealth University, Richmond, Virginia

**Keywords:** Alcohol and tobacco, alcoholism, cigarette smoking, nicotine dependence, emotional and psychiatric depression, comorbidity, agents for alcohol and other drug (AOD) concurrent mental disorders, cessation of AOD use (AODU), smoking cessation treatment, serotonin receptors, serotonin reuptake inhibitors, pharmacotherapy, bupropion, varenicline, topiramate, tricyclic antidepressant, nicotine replacement therapy, psychotherapy, cognitive behavioral therapy

## Abstract

Alcoholism and nicotine dependence share many neurobiological underpinnings; the presence of one drug can cause a person to crave the other. Depressive illness can complicate comorbid alcohol and nicotine dependence by exacerbating the negative affect encountered during attempts to abstain from one or both drugs. Given the morbidity and mortality associated with cigarette smoking, it is imperative to identify treatments to promote smoking cessation and address comorbid psychiatric conditions contemporaneously. Pharmacotherapeutic options demonstrating varying degrees of efficacy and promise in preclinical and clinical studies include nicotine replacement therapy (NRT), selective serotonin reuptake inhibitors (SSRIs), bupropion, varenicline, tricyclic antidepressants, and bupropion plus NRT. Topiramate has shown potential for promoting smoking cessation in alcoholics, although its safety in depressed patients has not been fully explored. The efficacy of medications for treating nicotine dependence is generally enhanced by the inclusion of behavioral interventions such as cognitive behavioral therapy. When group cohesion and social support are stressed, success rates increase among depressed smokers undergoing smoking cessation treatment. Additional treatment strategies targeting dually dependent individuals with comorbid psychiatric disorders, including special populations such as women and adolescents, await further investigation.

Cigarette smoking is the leading cause of preventable morbidity and mortality in the United States (McGinnis and Foege 1993). Fifty to 90 percent of people with mental illness or addiction also are nicotine dependent (Williams and Ziedonis 2004). Compared with the general population, alcohol-dependent or other mentally ill smokers have increased physical health consequences and mortality rates (Williams and Ziedonis 2004). Further, co-occurrence of substance abuse and depression is associated with greater impairment and worse treatment outcomes than either disorder alone (Brown et al. 2000). Therefore, smoking exacerbates the consequences of comorbid^1^ substance abuse and can complicate the course of psychiatric disorders.

People who are dependent on either alcohol or nicotine often are at increased risk for the other comorbid disorder. Smokers have two to three times greater risk for alcohol dependence than non-smokers (Breslau 1995), and about 80 percent of alcoholics also are dependent on nicotine (Hughes 1996). Indeed, recent research has suggested a neurobiological link between nicotine and alcohol dependence. For instance, alcohol and nicotine share a common neurobiological substrate involving the cortico-mesolimbic dopamine system, which is critical for expressing the positive reinforcing effects of these drugs (Hemby et al. 1997; Wise 1996). Preclinical studies also show that neurochemical interactions between alcohol and nicotine can augment the reinforcing effects of the combination (Soderpalm et al. 2000) and that the presence of one drug can trigger drugseeking behavior for the other (Lê et al. 2003).

Tobacco and alcohol seem to trigger similar central opioid peptide responses; therefore, both substances often are used as self-medication for comorbid affective disorder (Abrams et al. 1992; Hertling et al. 2005; Pomerleau and Pomerleau 1987). Thus, Currie and colleagues (2001) have suggested that people with a combined history of alcohol dependence and major depression are at high risk of using smoking as a means of mood enhancement. Craving for alcohol or nicotine is positively correlated with depression and anxiety, and alcohol-dependent patients often experience the urge to smoke in response to the discomfort associated with the urge to drink or to enhance their mood (Rohsenow et al. 1997).

Some practitioners might be reluctant to treat nicotine dependence in mental health settings if they think that it would depress mood and increase anxiety among patients trying to overcome other addictions or mental illness. Nevertheless, despite the belief that smoking cessation can trigger alcohol relapse among people dependent on both drugs, contemporary studies show that smoking cessation treatment does not cause abstinent alcoholics to relapse (Hughes and Callas 2003). Indeed, treatment that promotes smoking cessation among smokers within an alcoholdependent population might decrease the likelihood of relapse to drinking.

The clinical approach toward treating people with comorbid nicotine and alcohol dependence becomes more complicated among patients who also have a depressive illness. Such individuals might smoke or drink to relieve negative affective mood states such as depression and anxiety and therefore would be expected to be more difficult to treat. This article will review some of the treatments available to help patients with comorbid alcohol and nicotine dependence and depression, discuss the limitations of these treatments, and introduce some of the new treatment approaches that might lessen the challenge of treating this population.

**Figure:**
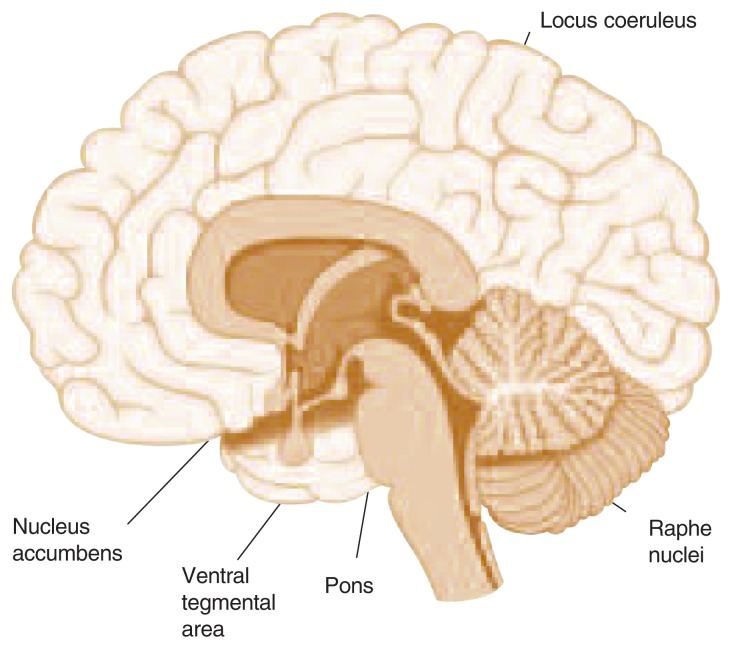
Parts of the brain involved in alcohol/nicotine dependence and psychiatric disorders.

## Basic Research

An understanding of the neurochemical mechanisms underlying the addictive properties of alcohol and nicotine is critical for the development of potential pharmacotherapies. As with other drugs of abuse, the reinforcing effects of both alcohol and nicotine appear to be mediated, at least in part, by dopaminergic projections in the cortico-mesolimbic system (Johnson 2004; Koob 2003; Samson and Harris 1992). Alcohol, both directly and indirectly, can increase excitatory cellular activation of dopaminergic cell bodies in the ventral tegmental area (VTA). This, in turn, leads to the facilitation of dopamine release in the nucleus accumbens (Brodie et al. 1999; Johnson 2005; Ortiz et al. 1995). The primary action by which alcohol increases dopamine in the nucleus accumbens appears to be via its effects on gamma-aminobutyric acid (GABA) neurons in the VTA (for a review, see Johnson 2004). Recent evidence, however, demonstrates that alcohol also can exert its reinforcing and dopamine-enhancing effects through activation of nicotinic acetylcholine receptors. In laboratory rats, chronic treatment with nicotine increases the reinforcing and dopamine-enhancing effects of alcohol, and these effects are blocked by the nicotinic receptor antagonist mecamylamine (for a review, see Larsson and Engel 2004). These results suggest that nicotinic acetylcholine receptors in the VTA might serve as a common substrate for alcohol–nicotine interactions.

Serotonin appears to play a critical role in mediating the reinforcing effects of alcohol and nicotine, and it has been implicated in the pathophysiology of various neuropsychiatric disorders, including depression. Both alcohol and nicotine stimulate the serotonergic system. Long-term use of alcohol and nicotine can, however, produce a hypo-serotonergic state that might trigger or worsen a depression. For instance, both chronic alcohol and nicotine administrations dose-dependently reduce the synthesis of tryptophan hydroxylase (the rate-limiting enzyme for serotonin synthesis) in the raphe nuclei, as evidenced by a diminution in the amount of serotonin- and tryptophan hydroxylase–positive cells identified by immunochemistry (Jang et al. 2002). It is reasonable, therefore, to propose that the pathogenesis of alcohol- and nicotine-induced mood disorders might involve alcohol- and nicotine-induced suppression of serotonin synthesis. Taken together, these data suggest that neuromodulation of serotonin and cortico-mesolimbic dopamine, particularly via manipulation of the nicotinic acetylcholine receptor, might reduce the reinforcing effects of both alcohol and nicotine. Thus, pharmacological agents that reduce the reinforcing effects of alcohol and nicotine by modulating these neurotransmitter systems might have potential therapeutic value for treating nicotine and alcohol dependence and comorbid depression in humans.

## Pharmacotherapeutic Approaches

Nicotine replacement therapy (NRT) in combination with psychotherapy or behavioral therapy is an effective treatment for nicotine dependence. Some research data also suggest that NRT might be beneficial in improving mood among abstinent depressed smokers (Cummings and Hyland 2005). Because the emergence of depressive symptoms during smoking cessation treatment is associated with failed quit attempts and increased probability of returning to smoking (Anda et al. 1990), alleviating such negative affective mood states is an important pharmacotherapeutic goal.

Although treating smokers with concurrent major depressive disorder requires the administration of antidepressants such as selective serotonin reuptake inhibitors (SSRIs), the utility of SSRIs in treating alcohol dependence has depended on clinical subtype. Among patients who develop problem drinking early in life, have a strong family history of alcoholism, and frequently exhibit impulsive behaviors (i.e., early-onset or type B alcoholics), SSRIs worsen drinking outcomes (Kranzler et al. 1996). In contrast, SSRIs improve drinking outcomes among patients who develop problem drinking later in life and do not have a family history of alcoholism or a personal history of impulsivity (i.e., late-onset or type A alcoholics) (Pettinati et al. 2000). Indeed, Johnson (2000) has proposed that the variation in expression of the molecular mechanism within the serotonin system might explain this differential response. Notwithstanding these findings, SSRI treatment appears to benefit alcohol-dependent patients with severe depression and suicidal ideation (Cornelius et al. 1997) but not to aid those with more moderate depressive symptoms and comorbid alcohol dependence (Pettinati et al. 2001). Further, even among depressed patients who are co-dependent on nicotine and alcohol, SSRI treatment alleviates the depressive mood but has little impact on the substance abuse–related outcomes (Torrens et al. 2005). Patients with a dual diagnosis (i.e., of depression and substance dependence) therefore need concomitant treatment of both disorders.

Bupropion is the only antidepressant that has been approved by the Food and Drug Administration (FDA) for treating nicotine dependence. Bupropion is a tricyclic antidepressant (TCA) that inhibits noradrenergic and dopamine uptake and, at high concentrations, inhibits the firing of noradrenergic neurons in the locus coeruleus (Ascher et al. 1995). Preclinical studies also show that bupropion might act as a noncompetitive nicotinic receptor antagonist (Slemmer et al. 2000), thereby reducing the reinforcing effects of nicotine. Slow-release bupropion aids smoking cessation among smokers with a history of major depression or alcoholism. Slow-release bupropion’s dose-dependent effect on smoking cessation observed among smokers with a history of depression was comparable to the effect observed among smokers with no history of depression or alcoholism (Hayford et al. 1999). However, because smokers with current depression and alcoholism (i.e., within the past year) were excluded from the study, these findings might be of limited significance. Unfortunately, bupropion has not been found to be effective for treating alcohol dependence.

Bupropion is the only TCA that has been approved by the FDA as a treatment for smoking dependence. Nevertheless, other nonapproved TCAs have been investigated for smoking cessation and are considered to be second-line treatments. Nortriptyline, a TCA that has both dopaminergic and adrenergic enhancing effects, has shown efficacy in treating nicotine dependence independently of depression history and can reduce smoking cessation–related negative affect, which can be a trigger for relapse (Hall et al. 1998). Results from two separate research studies showed that TCAs such as desipramine (Mason et al. 1996) and imipramine (McGrath et al. 1996) also reduced depressive symptoms among alcoholics with comorbid depression. Although desipramine also demonstrated an effect to reduce drinking, this occurred at doses higher than that approved by the FDA, and there was increased risk of toxicity (Mason et al. 1996). TCAs can, however, produce several unpleasant adverse effects that limit their utility as antismoking agents. These include reducing the pleasurable effects of smoking related to its consumption by inducing drowsiness, making smoking more hazardous by increasing the potential for cardiotoxicity, and increasing the difficulty of smoking cessation by inducing weight gain. A recent systematic meta-analytic review concluded that antidepressant medication only exerts a modest beneficial treatment effect among patients with combined substance use and depressive disorders. It is not a stand-alone treatment; concurrent therapy aimed at directly treating the addiction also is indicated (Nunes and Levin 2004).

The FDA recently approved a second medication, varenicline, as an aid to smoking cessation. Varenicline is a selective α4 β2 partial nicotinic receptor agonist that, in the presence of nicotine, acts as a relative antagonist and diminishes nicotine’s reinforcing effects. In two recent trials, varenicline administration resulted in quit rates significantly higher than those achieved among placebo recipients (Nides et al. 2006; Oncken et al. 2006). Indeed, the results of one of these studies suggest that varenicline might be more clinically effective than bupropion (Nides et al. 2006). For a review, see Johnson (2006).

Combining NRT with non-NRT pharmacotherapeutic treatments appears promising for patients with comorbid depression and nicotine dependence. For instance, combining the nicotine patch with bupropion increases abstinence rates up to 58 percent, compared with bupropion alone (49 percent), patch alone (36 percent), or placebo (23 percent) (Johnston et al. 1999). Other studies have reported similar trends. For example, bupropion plus the transdermal nicotine patch increased 6-month, self-reported abstinence rates up to 34 percent, compared with bupropion alone (28 percent) and patch alone (15 percent) (Gold et al. 2002). In another study, bupropion combined with the nicotine patch resulted in higher 12-month abstinence rates (36 percent) compared with nicotine patch alone (16 percent), bupropion alone (30 percent), or placebo (16 percent) (Jorenby et al. 1999).

Topiramate is an anticonvulsant drug, with several mechanisms of action, that diminishes cortico-mesolimbic dopamine by facilitating GABAergic activity while inhibiting glutamatergic activity (for a review, see Johnson 2004). Johnson and colleagues (2003) have shown that up to 300 mg/day of topiramate, compared with placebo, significantly increases abstinence from alcohol among alcohol-dependent patients receiving only brief behavioral compliance enhancement treatment. In a subset of participants from the same trial, topiramate recipients compared with placebo recipients were significantly more likely to become abstinent from smoking (odds ratio = 4.46; 95 percent CI 1.08–18.39; *p* = 0.04). Interestingly, reductions in smoking were positively associated with drinking decreases for the topiramate group but not for the placebo group. These results suggest that topiramate might have specific antismoking effects. Topiramate did not alter mood. Study participants who received topiramate, compared with those who received the placebo, experienced a significant weight reduction (i.e., 20 [44 percent] topiramate recipients experienced weight loss compared with 9 [18 percent] placebo recipients; *p* = 0.008) (Johnson et al. 2005). Taken together, these results demonstrate topiramate’s potential as a safe and promising medication for treating alcoholdependent smokers. Topiramate’s ability to induce weight loss might counter a treatment barrier reported by some in smoking cessation treatment—weight gain (Jeffery et al. 2000). A double-blind, placebo-controlled clinical trial testing topiramate for the treatment of anger and depressive symptoms among mildly to moderately depressed women in Germany showed efficacy in primary outcome measures, which included significant reductions on the Hamilton Depression Rating Scale, the State-Trait Anger Expression Inventory, the Test of Attention, and the SF-36 Health Survey (Nickel et al. 2005). Nevertheless, the type of alcohol-dependent smoker who responds best to topiramate needs to be elucidated more clearly, and research is needed to determine the safety of topiramate in treating comorbid alcohol- and nicotine-dependent patients with current or a past history of depressive illness.

## Psychotherapeutic Intervention

Two psychological theories have been proposed as being related to the relationships among depression, alcoholism, and smoking behavior. First, drug use (including that of nicotine and alcohol) might be motivated by a person’s expectation of the outcome, such as a decrease in negative affect and tension. If such a person is also depressed, there might be an even greater motivation to alleviate dysphoria or anxious mood. Second, depressed smokers with or without alcohol dependence might have less self-efficacy and, therefore, more difficulty becoming abstinent than their nondepressed counterparts.

These models of self-medication and learned helplessness have received empirical support in research with nicotine- and alcohol-dependent populations. For example, smokers who identify the prevention of negative affect as their primary reason for smoking are more likely to fail in quit attempts. When smokers become depressed, the course and prognosis of smoking cessation become intertwined with the pathophysiology of depression. For instance, smokers with a history of major depressive disorder are more likely to experience depressed mood during nicotine withdrawal (Breslau et al. 1991) and are at greater risk for developing recurrent episodes of major depression than are nonsmokers (Glassman et al. 2001). Depressed smokers also are more likely than nondepressed smokers to report deficits in coping resources, to adhere to the components of smoking cessation treatment, and to use cigarettes for ameliorating negative affect (Kinnunen et al. 1996). Thus, psychosocial interventions for depressed smokers with or without alcohol use disorders might be more effective if they were focused on treating depressive symptoms simultaneously with the smoking cessation treatment. This would enable such individuals to learn healthy coping strategies, including affect regulation and stress management.

Indeed, in one of the few studies of its kind, Patten and colleagues (2002) examined the effect of depressive symptoms on smoking abstinence and treatment adherence among smokers with a past history of alcohol dependence. They found that smokers with a history of alcohol dependence who also reported high levels of depressive symptoms were more likely to be abstinent from smoking at the end of treatment and at follow-up than those with low depression scores. The authors concluded that similar to research in the nonalcoholic literature (Zelman et al. 1992), smokers who demonstrate a vulnerability to negative affect—and who might use drugs to regulate mood—benefit more from a mood management–specific therapy to address their depression in addition to smoking cessation treatment.

Nevertheless, the necessity of incorporating mood management skills training for smokers with a history of depression has not been established. For instance, Hall and colleagues (1994, 1998) found that mood management skills training for smokers with a history of major depressive disorder was most effective when there was more frequent therapist contact time than in the control group. However, no significant effect of mood management was observed when both the target and control groups received equal therapist time (Hall et al. 1996). Mood management did not attenuate postcessation increases in depression among smokers with a past history of depression.

Other types of psychotherapy might be more effective than mood management in treating people with comorbid nicotine dependence and depression. Brown and colleagues (2001) found that heavy smokers with a history of major depressive disorder had better outcomes when cognitive behavioral therapy (CBT) for depression was incorporated into a standard smoking cessation treatment. Although CBT for depression did not decrease depressive symptoms prior to or after smoking cessation, it did prevent the expected rise in such symptoms following smoking cessation. In a recent review of combination treatment for nicotine dependence, Ingersoll and Cohen (2005) reported that treatments combining a behavioral component with a first-line pharmacotherapeutic agent enhanced smoking cessation rates more than either alone. They concluded that some forms of psychosocial treatment, “such as those based on principles of effective brief therapies, and using techniques of CBT, can enhance the gains achieved with first-line pharmacotherapies for smoking cessation in general populations of smokers.”

Depressed women appear to be particularly responsive to certain types and modalities of psychotherapy administration. For instance, Ginsberg and colleagues (1997) have reported that group CBT was an effective and important part of a multicomponent (CBT plus nicotine gum) smoking cessation program for women with and without a history of depression. In that study, standard smoking cessation group CBT included learning strategies for smoking cessation, goal setting for nicotine fading, increased social support, problem-solving skills training, and, finally, weaning off gum after quitting nicotine. This type of CBT, which emphasizes group cohesion and social support, helped to maintain adherence, thereby promoting effective treatment and smoking cessation regardless of whether there was a history of depression. One caveat to this study is that the authors did not evaluate the impact of the treatment on depressive symptoms.

Alcohol dependence complicates the pathophysiology, course, and treatment outcome of depressed smokers. Patten and colleagues (2002) found that smokers with a history of alcohol dependence, who also reported high levels of depressive symptoms, were more likely to be abstinent from smoking at the end of treatment and at follow-up than those with low depression scores. Thus, Zelman and colleagues (1992) have proposed that smokers who are vulnerable to negative affect— and who use drugs to regulate mood—might benefit more from a mood management–specific therapy to address their depression in addition to smoking cessation treatment.

In summary, incorporating a behavioral intervention with pharmacotherapy for smoking cessation increases the success rate, perhaps because of an improved adherence to treatment. CBT that emphasizes group cohesion and social support appears to be beneficial in maintaining treatment adherence among depressed smokers within smoking cessation treatment and helps to prevent the expected rise in depressive symptoms upon quitting. The added benefit of teaching mood management skills has, however, not been established in the treatment of depressed smokers with or without alcohol dependence.

## Gender- and Age-Specific Issues Associated With Treatment Outcome

Rates of smoking are much higher among people who have alcohol problems and a history of depressive illnesses than among people without those disorders (Currie et al. 2001). Depression also is associated with earlier relapse in treated teens with alcohol use disorder (Cornelius et al. 2004) as well as in adult alcoholics (Greenfield et al. 1998). Among smokers, females tend to have a worse problem abstaining from nicotine than their male counterparts (Perkins et al. 1999). There are, at least, two possible explanations for this gender difference. First, among smokers, women are more concerned than men about weight gain following smoking cessation. Second, because women are more prone to affective disorder, they tend to have greater negative affect associated with smoking cessation. Nevertheless, research in this area is still developing, and little is known about the gender- and agespecific issues associated with treatment outcome among depressed smokers who also are alcohol dependent.

## Conclusions

People with concurrent mental disorders, such as major depression and alcohol and nicotine dependence, are increasingly prevalent in clinical practice and generally have poor response to treatments, which can be costly. Nevertheless, there is growing evidence that contemporaneous treatment for depressive disorder and smoking cessation is preferable to treatment of either condition alone, even in the presence of alcohol dependence. In such cases, combining different pharmacological agents in conjunction with CBT or other psychotherapies appears to be the preferred mode of treatment. New medications such as topiramate that could treat both alcohol and nicotine dependence might simplify the use of combination therapies if there is a concomitant depressive illness. The development of more specific pharmacological strategies targeting the populations most likely to respond—or working at different phases of the disorder(s)—is in its infancy. Among the various psychotherapies, CBT that emphasizes group cohesion and social support appears to be particularly useful for treating depressed smokers with or without alcohol dependence. The necessity of teaching mood management skills among people with nicotine and alcohol dependence who are also depressed has not been established. New knowledge is needed to develop treatments that might benefit special populations, including women and teenagers.

## Footnotes

1 For a definition of this and other technical terms used in this article, please see the glossary, p. 220.

## GLOSSARY

Adrenergic: Relating to *neurons* that are activated by, characteristic of, or secreting adrenaline or substances with similar activity.
Affect disorder/Affective disorder: Mental disorders controlling mood.
Antagonist: An agent that blocks or reverses the actions or effects of another agent (e.g., a drug that blocks the effects of a *neurotransmitter*).
Brief behavioral compliance enhancement treatment: Psychosocial adherence enhancement program that emphasizes the importance of medication compliance in changing an alcoholic’s drinking behavior.
Cardiotoxicity: A condition where there is damage to the heart muscle or its function.
Central opioid peptides: Molecules in the brain that modify the actions of other *neurotransmitters*. By altering the electrical properties of their target *neurons*, thereby making these neurons more difficult to excite, opioid peptides can influence the release of various neurotransmitters. As a result of this modulation, opioid peptides can—among other functions— induce pain relief and euphoria as well as affect certain behaviors, including alcohol consumption.
Comorbidity: In general, comorbidity refers to the co-occurrence or overlap of two or more psychiatric disorders.
Cortico-mesolimbic dopamine system: A circuit in the brain through which *dopamine* is transmitted.
Dopamine: An excitatory *neurotransmitter* that plays a role in the reward system in the brain and possibly in the reinforcing properties of alcohol.
Dopaminergic: Relating to *neurons* or nerve fibers that release *dopamine.*
Dopaminergic projections: Means by which *dopamine* is transmitted between cells.
Dysphoria: Generally characterized as a feeling of emotional and/or mental discomfort, restlessness, malaise, and depression.
Excitatory: Increasing the capacity of a nerve cell to respond to stimuli.
GABAergic: Relating to *neurons* or nerve fibers that release *gamma-aminobutyric acid* (GABA).
Gamma-aminobutyric acid (GABA): An inhibitory *neurotransmitter* the actions of which are influenced by alcohol; may play a role in alcohol withdrawal.
Glutamatergic: Relating to *neurons* or nerve fibers that release the *neurotransmitter* glutamate.
Hypo-serotonergic state: Lacking *serotonin*.
Locus coeruleus (see graphic): A pair of identical nuclei (clusters of *neurons*) in the *pons* from which all brain connections using norepinephrine arise.
Neurobiological substrate: Substance in the brain upon which enzymes act to influence emotional states and disorders.
Neuromodulation: The action of neuromodulators, substances similar to *neurotransmitters* that are released by *neurons* and convey information to adjacent or distant neurons. Neuromodulators indirectly affect the excitation or inhibition of neurons by changing the way in which neurons react to neurotransmitters.
Neuron: A nerve cell.
Neurotransmitter: A chemical messenger released by an excited or stimulated *neuron.* A neurotransmitter binds to a *receptor* on an adjacent neuron, usually triggering a series of chemical and electrical changes in the second cell.
Nicotinic acetylcholine receptors: *Receptors* that recognize and bind with both nicotine and the *neurotransmitter* acetylcholine.
Nicotinic receptor antagonist: A compound that blocks nicotine *receptors*.
Noradrenergic: Stimulated by or releasing the *neurotransmitter* norepinephrine.
Nucleus accumbens (see graphic): A brain structure affected by many drugs of abuse and implicated in the rewarding properties of addictive drugs.
Pathogenesis: The mechanism by which certain etiological factors cause disease.
Pons (see graphic): A broad mass of nerve fibers that forms the central portion of the brainstem. The pons participates in control of respiration and coordination of muscular activity.
Preclinical studies: Studies used to test experimental drugs in the test tube or in animals; the testing that occurs before trials in humans may be carried out.
Raphe nuclei (see graphic): A moderate-sized group of nuclei (clusters of *neurons*) found in the brainstem that releases *serotonin* to the rest of the brain.
Rate-limiting enzyme: The slowest step in the creation of a molecule, and often the most important, because it requires additional energy and is highly regulated. The rate-limiting enzyme can have the biggest effect on the final product, so if something is wrong with it, the effect of this malfunction will translate down the chain to the end product.
Receptor: A protein usually found on the surface of a *neuron* or other cell that recognizes and binds to *neurotransmitters* or other chemical messengers.
Selective α4 β2 partial nicotinic receptor agonist: An inhibitor of *dopaminergic* activation produced by smoking that simultaneously provides relief from the craving and withdrawal.
Selective serotonin reuptake inhibitor: A class of antidepressants used in the treatment of depression, anxiety disorders, and some personality disorders.
Serotonergic: Relating to the *neurotransmitter serotonin*.
Serotonin: A *neurotransmitter* involved in many functions, including mood, appetite, and sensory perception. A lack of serotonin in the brain is thought to be a cause of depression.
Ventral tegmental area (see graphic): The midbrain region containing *dopamine* cell bodies that project to various parts of the forebrain, including the *nucleus accumbens.*
